# Non-invasive Stenotic Renal Artery Haemodynamics by *in silico* Medicine

**DOI:** 10.3389/fphys.2018.01106

**Published:** 2018-08-17

**Authors:** Aikaterini Mandaltsi, Andrii Grytsan, Aghogho Odudu, Jacek Kadziela, Paul D. Morris, Adam Witkowski, Timothy Ellam, Philip Kalra, Alberto Marzo

**Affiliations:** ^1^INSIGNEO Institute for in silico Medicine, University of Sheffield, Sheffield, United Kingdom; ^2^Mechanical Engineering Department, University of Sheffield, Sheffield, United Kingdom; ^3^Division of Cardiovascular Sciences, University of Manchester, Manchester, United Kingdom; ^4^Salford Royal Hospital NHS Foundation Trust, Salford, United Kingdom; ^5^Department of Interventional Cardiology and Angiology, Institute of Cardiology, Warsaw, Poland; ^6^Department of Infection, Immunity and Cardiovascular Disease, University of Sheffield, Sheffield, United Kingdom; ^7^Department of Cardiology, Sheffield Teaching Hospitals NHS Foundation Trust, Sheffield, United Kingdom

**Keywords:** computational fluid dynamics, fractional flow reserve, precision medicine, cardiovascular modeling, non-invasive diagnosis, renal artery haemodynamics, *in silico* medicine

## Abstract

**Background:** Measuring the extent to which renal artery stenosis (RAS) alters renal haemodynamics may permit precision medicine by physiologically guided revascularization. This currently requires invasive intra-arterial pressure measurement with associated risks and is rarely performed. The present proof-of-concept study investigates an *in silico* approach that uses computational fluid dynamic (CFD) modeling to non-invasively estimate renal artery haemodynamics from routine anatomical computed tomography (CT) imaging of RAS.

**Methods:** We evaluated 10 patients with RAS by CT angiography. Intra-arterial renal haemodynamics were invasively measured by a transducing catheter under resting and hyperaemic conditions, calculating the translesional ratio of distal to proximal pressure (Pd/Pa). The diagnostic and quantitative accuracy of the CFD-derived virtual Pd/Pa ratio (vPd/Pa) was evaluated against the invasively measured Pd/Pa ratio (mPd/Pa).

**Results:** Hyperaemic haemodynamics was infeasible and CT angiography in 4 patients had insufficient image resolution. Resting flow data is thus reported for 7 stenosed arteries from 6 patients (one patient had bilateral RAS). The comparison showed a mean difference of 0.015 (95% confidence intervals of ± 0.08), mean absolute error of 0.064, and a Pearson correlation coefficient of 0.6, with diagnostic accuracy for a physiologically significant Pd/Pa of ≤ 0.9 at 86%.

**Conclusion:** We describe the first *in silico* estimation of renal artery haemodynamics from CT angiography in patients with RAS, showing it is feasible and diagnostically accurate. This provides a methodological framework for larger prospective studies to ultimately develop non-invasive precision medicine approaches for studies and interventions of RAS and resistant hypertension.

## Introduction

Renovascular disease is characterized by unilateral or bilateral renal artery stenosis (RAS). In Western populations 90% of RAS is caused by atherosclerotic renal artery stenosis (ARAS), and 10% by fibromuscular dysplasia (FMD) (Textor, 2017). ARAS affects 7% of North Americans aged over 65 years (Hansen et al., 2002), and the incidence is rising due to aging, obesity, diabetes, and hypertension (Kalra et al., 2005). FMD affects 0.4% of the population and is seen in younger patients (Slovut and Olin, 2004). Reduced renal perfusion causes progressive chronic kidney disease (CKD) and drives neurohormonal activation with subsequent resistant hypertension, end-stage kidney disease, and death (Textor, 2017). Major trials testing efficacy of reperfusion by angioplasty and stenting demonstrated no benefit beyond drug therapy (antihypertensives and statins) (Wheatley et al., 2009; Riaz et al., 2014). However, those trials recruited patients with physiologically mild ARAS and less severe CKD. This group are less likely to derive benefit from revascularisation compared with those with more severe ARAA and CKD (Hagemann et al., 2017). There is therefore a need for less invasive method to determine the physiological significance of ARAS. Moreover, neutral outcomes might have been exemplified due to patients with: median stenoses of no less than 70%, preserved renal function, and low annual mortality (Ritchie et al., 2014). It is acknowledged that a visually measured RAS diameter for the assessment of the lesion’s severity has a poor correlation to quantitative methods (Kadziela et al., 2016). Moreover, multiplanar angiographic assessment has a poor correlation with physiological assessment of the pressure and flow dynamics (Drieghe et al., 2008). The dissociation is attributed to two-dimensional angiographic views that ignore: complex vessel geometry, lesion length, radiolucent atherosclerotic plaques, collateral circulation, microvascular remodeling, and renal parenchymal injury both distal to the stenosis and in the contralateral kidney (Johnson et al., 2013).

The Cardiovascular Outcomes in Renal Atherosclerotic Lesions (CORAL) trial was initially designed to select haemodynamically severe ARAS cases, but eligibility criteria were expanded due to slow recruitment (Clinical Trials Identifier: NCT0081731^1^). This was related to the added complexity, risk, and cost of invasive haemodynamic measurements in ARAS which are not routine clinical practice. Recent subgroup analysis of the CORAL trial found no benefit of revascularization amongst those with more haemodynamically severe ARAS (Murphy et al., 2015). Hence the potential of RAS haemodynamics to permit precision medicine through physiologically guided trials of revascularization remains uncertain and further studies are hampered by the need for invasive measurements with associated risks.

The same haemodynamic and clinical considerations are apparent in the context of coronary artery disease. In 2007, the landmark COURAGE trial failed to demonstrate prognostic benefit from percutaneous coronary intervention (PCI) in stable coronary artery disease (Boden et al., 2007), whereas subsequent studies did demonstrate prognostic benefit when patients with demonstrable ischaemia, i.e., physiologically significant lesions were specifically targeted (Shaw et al., 2008). More recently, a number of studies have demonstrated the superiority of physiological over anatomical assessment of CAD (De Bruyne et al., 2014).

*In silico* medicine describes the use of computational simulations in the diagnosis, treatment or prevention of disease. A robust *in silico* technique for the non-invasive assessment of the haemodynamic severity of RAS would enable targeted trial recruitment and therapeutic intervention, to those most likely to derive benefit. Better characterisation of RAS lesion severity could also reduce the sample size required for trials of novel interventions. An *in silico* application, called VIRTUheart, has been developed at the University of Sheffield to compute the physiological significance of coronary artery disease from angiography using CFD modeling (Morris et al., 2013). The aim of this proof-of-concept study was to develop and validate a similar CFD model to predict RAS haemodynamics from computed tomography (CT) imaging.

## Materials and Methods

### Patients

Demographic, imaging and haemodynamic data from 10 patients with 11 stenoses (one patient had bilateral RAS) were provided by the Department of Interventional Cardiology and Angiology (Warsaw, Poland). The patients were both female and male, aged between 40 and 79 years of age. **Table 1** presents the clinical profiles for the anonymised patient data set. Ethics approval for sharing and analyzing retrospective anonymised patient data was obtained from the local Bioethics Committee at the Institute of Cardiology in Warsaw. Patient data was fully anonymised prior to data sharing and analysis. We collected CT and invasive angiographic imaging data from consenting patients with hypertension and RAS. Patients with prior contrast nephropathy, severe valvular disease, New York Heart Association (NYHA) III-IV heart failure or estimated Glomerular Filtration Rate (eGFR) below 30 mL/min were excluded. Serum creatinine was measured and eGFR was calculated by the modification of diet in renal disease (MDRD) 4-variable equation for each patient (Levey et al., 2006). eGFR reflects kidney function and may also reflect distal vascular resistance which is important for the computational model.

**Table 1:** Patient Characteristics.

Patient no.	Age (yrs)	Gender	Stenotic Side	Condition	Diameter stenosis (%)	eGFR (mL/min/1.73m^2^)
1	74	Female	Left	ARAS	72	62
2	65	Female	Left	ARAS	46	46
3	64	Male	Left	ARAS	72	72
4	79	Male	Right	ARAS	20	43
5	58	Male	Left	ARAS	42	86
6	57	Female	Right	FMD	43	87
7	49	Female	Left	ARAS	53	125
8	74	Male	Left	ARAS	76	33
9	72	Female	Right	ARAS	67	65
10	40	Female	Both	FMD	n.a.	102

*Age was at the time of the CT image acquisition. ARAS, atherosclerotic renal artery stenosis; FMD, fibromuscular dysplasia; eGFR, estimated Glomerular Filtration Rate. Diameter stenosis values were calculated using the formula DS = 100-(MLD/RLD)^∗^100, where DS is diameter stenosis, MLD is minimum lumen diameter, RLD is reference lumen diameter*.

### CT Renal Angiography

**FIGURE 1:**
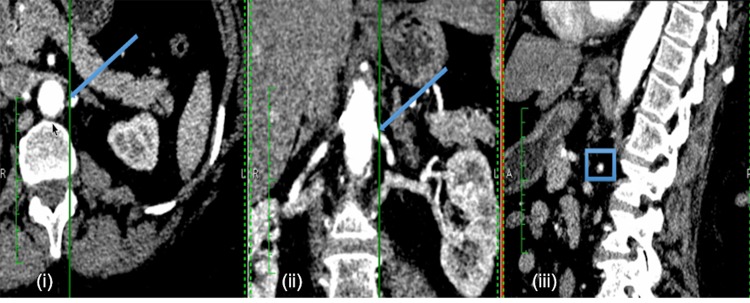
Example of patient-specific CT images. Example CT images of each anatomical orientation for Patient 2: axial **(i)**, coronal **(ii)**, and sagittal **(iii)**. The arrows in the axial and coronal slices, and the box in the sagittal slice indicate the approximate location of the renal stenosis for the specific case.

Computed tomography renal angiography was performed with a 64-detector CT scanner (Somatom Sensation Cardiac 64; Siemens, Erlangen, Germany). ARAS patients had CT images acquired as a part of the PREFFER study (Kadziela et al., 2011). The two FMD patients underwent imaging as per standard clinical practice. A minimum of 100 CT slices in axial, coronal, and sagittal orientations were acquired with in-plane resolution by pixel spacing of 0.59 to 0.83 mm. A typical example is shown in **Figure 1**.

### Invasive Haemodynamic Measurements

Heparin (4000–5000 IU) was administered to maintain adequate anticoagulation during the procedure. Distal pressure (Pd) was measured with a 0.014˝ Pressure Wire 5 (Radi Medical Systems, Sweden) and proximal pressure (Pa) was measured from the guiding catheter tip. During pressure measurements, the tip was disengaged from the ostium to avoid pressure damping. The translesional ratio Pd/Pa was calculated as the ratio of mean Pd to mean Pa. This *in vivo* measured Pd/Pa ratio (mPd/Pa) is a standard measure to evaluate the haemodynamic significance of an arterial stenosis (Subramanian et al., 2005). The hyperaemic renal Fractional Flow Reserve (rFFR) was calculated in the same way after the administration of 30 mg of papaverine into the renal artery distal to the stenosis via a 3F multifunctional catheter.

## Computational Workflow

Our computational workflow segmented and reconstructed the patient-specific three-dimensional arterial geometries from the CT images. CFD analysis was used to simulate the translesional haemodynamics. The computed results were used to calculate the ‘virtual’ Pd/Pa (vPd/Pa) which was validated against the invasively measured Pd/Pa (mPd/Pa).

### Volume Segmentation

The workflow’s first step involved segmenting a volume from the available CT images. For that purpose the non-parametric geodesic active regions (GAR) method was implemented, following the algorithm presented in (Hernandez and Frangi, 2007). This segmentation model was tested and made available through the application @neufuse, initially developed as part of the ‘@neurIST project’^2^.

### Computational Fluid Dynamics

**FIGURE 2:**
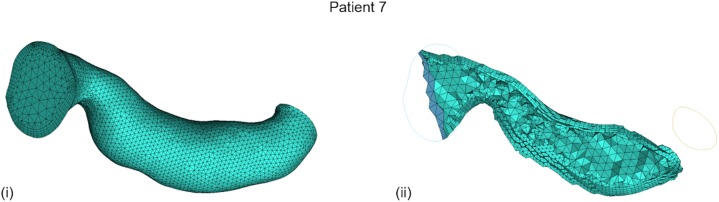
Typical volume mesh for the CFD simulations: **(i)** the mesh elements on the wall and on the inlet cross-section, and **(ii)** the tetrahedral elements inside the volume and the prismatic layers near the wall on a cut plane can be observed.

CFD was implemented on the ANSYS^®^-CFX^TM^ (ANSYS Canonsburg, United States) simulation software: a volumetric mesh was created, the flow’s boundary conditions were set, the flow was solved on a Navier–Stokes-based solver, and the flow solution was post-processed for the vPd/Pa estimation. The method from Marzo et al. (2009) was adopted for the creation of the volumetric mesh: an octree approach was implemented with finer grids at the wall, tetrahedral elements inside the volume, and five layers of prismatic elements adjacent to the wall (for better accuracy of the velocity gradient). An average mesh density of approximately 200 elements per cubed millimeter was chosen. An example volume mesh cross-section is presented in **Figure 2**.

Typical renal boundary flow conditions were chosen for the CFD on the renal arterial segments. The 1D model of the systemic arterial tree by Reymond et al. (2011) was utilized, which provides pressure and flow values (at rest) over a heart cycle for various points within the renal vasculature, based on the healthy state. Based on these data, an inlet velocity (with a plug flow velocity profile) and an outlet peripheral resistance were set. Peripheral resistance is defined as the ratio of the pressure drop from the point of measurement to the capillary level along that branch to the flow passing through the point of measurement. It describes the resistance encountered by the blood as it flows through the systemic arterial system and represents the effect of downstream microcirculation (of smallest arteries and arterioles) (Bott, 2014). For the steady flow simulations of the current study we calculated an average from Reymond’s values over the heart cycle for the pressure and flow boundary conditions in the renal arteries. The ultimate vPd/Pa calculation is thus a time-averaged parameter extracted from the time-dependent boundary data. It should be noted that, under the aforementioned boundary conditions, hyperaemic haemodynamics was infeasible and the study focuses on resting flow conditions.

Additionally, we compared the vPd/Pa estimations when the geometry included the aortic and contralateral renal geometry, and when it did not. Reymond’s boundary conditions are not representative of our patient data set, consisting of old and diseased subjects. When the geometry only includes the renal arterial segment, a realistic flow is imposed by the inlet boundary condition and therefore this limitation is overcome. However, when the inlet velocity boundary condition is set on the aorta, Reymond’s measurements underestimate the resistance of renal artery with stenosis, resulting in much higher resistance to blood flow entering the renal artery, which in turn results in insufficient blood flow into the renal artery.

**FIGURE 3:**
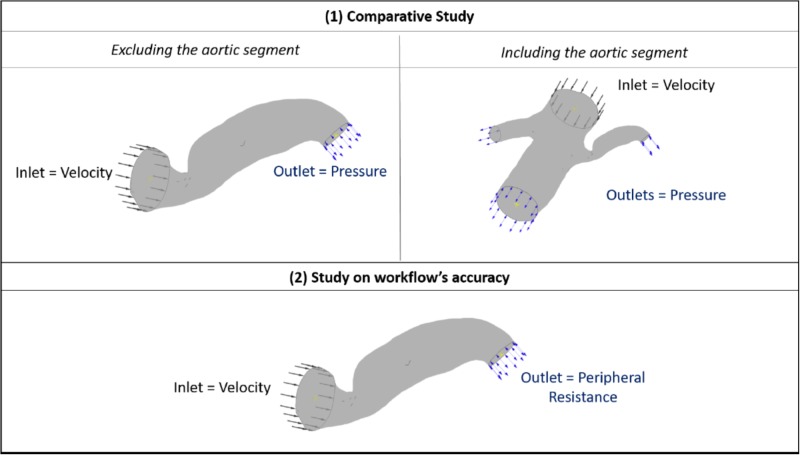
Boundary flow conditions for the set of simulations. The image summarizes the boundary flow conditions applied on the computational simulations: **(1)** for the comparative study between including and excluding the aortic segment (top row) and **(2)** for testing the accuracy of the workflow (bottom row) across the patient data set. The case of Patient 7 is used here for illustration purposes.

Therefore, for the purposes of this comparative part of our study (patient 7 and 10), we fine-tuned the outlet pressure in order for the blood flow into the renal artery to reach expected generic levels. A direct comparison between the two geometric segments was then possible by setting the boundary flow conditions, as follows: (i) for the cases including the aorta, a generic inlet aortic velocity boundary, and adjusted outlet stenotic renal, contralateral renal and downstream aortic pressure were defined, and (ii) for the cases excluding the aorta, a generic inlet renal velocity and the same adjusted outlet renal stenotic pressure as with case (i) were defined. The boundary flow conditions for the comparative study and for the aforementioned simulations on the complete data set are illustrated in **Figure 3**.

**FIGURE 4:**
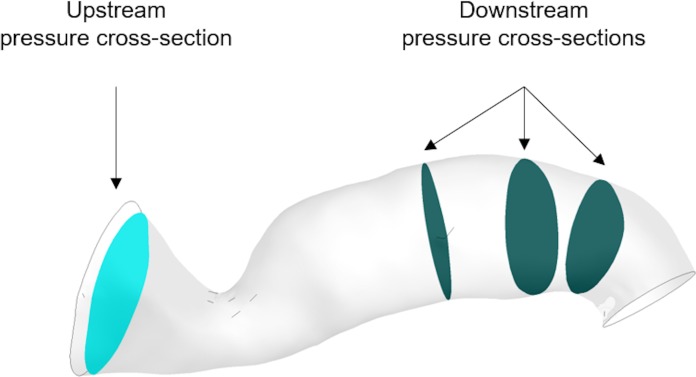
Downstream and upstream pressure monitoring cross-sections.

The program solves the steady incompressible Navier–Stokes momentum equations in combination with the continuity equation. The flow solution follows the implicit finite volume discretisation method for the numerical approach. The blood flow is modeled as a Newtonian fluid of viscosity at μ = 0.0035 Pa s and constant density ρ = 1066 kg m^-3^, and the arterial wall is assumed to be rigid (Ferziger and Peric, 2002). The computer used for the simulations was an Intel(R) Xeon(R) X5690 CPU @ 3.47 GHz x 12, 24.0 GB RAM, 64-bit OS (Windows 7). The steady flow simulations took on average 2 min to solve. The general clinical protocol indicated that the proximal pressure was measured furthest from the stenosis and close to the opening to the aorta, whereas the distal pressure was measured 10–20 mm downstream of the stenosis. We defined three downstream cross-sectional areas within this range and calculated the average pressure across each of them. Downstream pressure was then simply defined as the arithmetic average of the three. For the upstream pressure, an additional cross-sectional area was carefully chosen to exclude pressure extremes resulting from the Bernoulli effect due to the proximity of the stenosis to the inlet boundary, and the average pressure across it was calculated. vPd/Pa was then estimated as the ratio of downstream to upstream pressure (**Figure 4**).

### Statistical Analysis

We follow the statistical analysis of (Morris et al., 2013) for the diagnostic and quantitative accuracy of our workflow. Consensus guidelines for RAS stenting (Parikh et al., 2014) and research on the criteria for renovascular hypertension due to RAS (De Bruyne et al., 2006) indicate a Pd/Pa of ≤ 0.9 as physiologically significant. On that basis, the diagnostic accuracy was evaluated by calculating the sensitivity, specificity, positive predictive value (PPV), negative predictive value (NPV) and overall accuracy for our results, with the binomial test’s 95% confidence intervals (CIs). The correlation between mPd/Pa and vPd/Pa data was visualized and assessed through a diagram plot. Additionally, agreement was measured by the mean difference and absolute error between measured and virtual values, and the standard deviation of the differences were computed in order to illustrate the quantitative accuracy of the workflow in a Bland–Altman plot.

## Results

### Volume Segmentation

**FIGURE 5:**
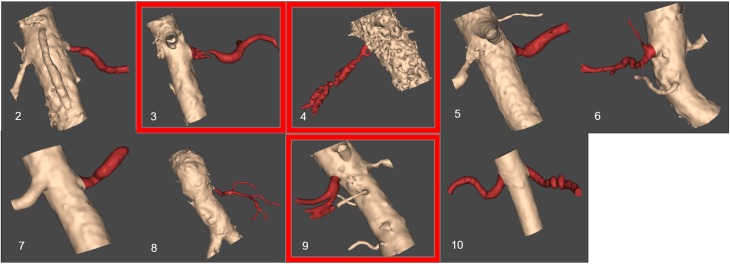
Resulting segmented patient renal geometries. Segmented volumes of renal arteries: unsuccessful segmentation examples based on the provided CT images are indicated by a red frame. The number for each image corresponds to patient number in **Table 1**.

**Figure 5** presents the reconstructed geometries, following a process of manual correction whereby the region of interest was separated from local tissue artifact. Three of the CT scans were of insufficient quality for vessel reconstruction (indicated by a red frame in **Figure 5**) and were therefore excluded from analysis. For an additional case, the number of CT slices were insufficient in order for the segmentation tool to process them. The successfully segmented stenosed renal arteries include the descending aorta and the contralateral renal artery. Patients whose renal arteries show multiple stenoses, for example Patient 10, were suffering from FMD.

### Simulations

The following sections present the results from our study, including a quantitative comparison to assess the accuracy of the vPd/PA against the mPd/Pa from the simulations in the complete dataset of 7 RASs.

### Comparative Study With and Without the Aorta

**Table 2** shows the calculations of vPd/Pa for the two patients of our comparative study: Patient 7 (one stenosis), and Patient 10 (double stenosis). A percentile differences in vPd/Pa is estimated between the computations when the aortic geometry is included and when it is not, demonstrating a maximum difference of 1.28% for the left RAS of Patient 10. The remaining two cases present a difference of less than 1%. **Table 2** (last column) also shows the variability in the values of downstream pressure extracted from the three different post-stenotic locations, showing a direct correlation between pressure variability and anatomical complexity (**Figure 5**).

**Table 2:** Comparative study between including and excluding the aortic geometry in CFD.

Patient no.	vPd/Pa (incl. aorta)	vPd/Pa (excl. aorta)	difference [%]	Pd [Pa]
*7*	0.847	0.865	2.1	13374 ± 69
*10 (Right)*	0.829	0.862	3.8	12806 ± 8
*10 (Left)*	0.852	0.853	0.1	13037 ± 169

*Quantitative differences of vPd/Pa when implementing CFD with geometries that include and exclude the aortic segment, for Patients 7 and 10 (Patient 10 presents a renal stenosis on both the right and the left renal artery). The last column reports the arithmetic average and standard deviation (SD) of the downstream pressure (Pd) calculated at three different locations for the case without the aorta only*.

### Quantitative Accuracy

**FIGURE 6:**
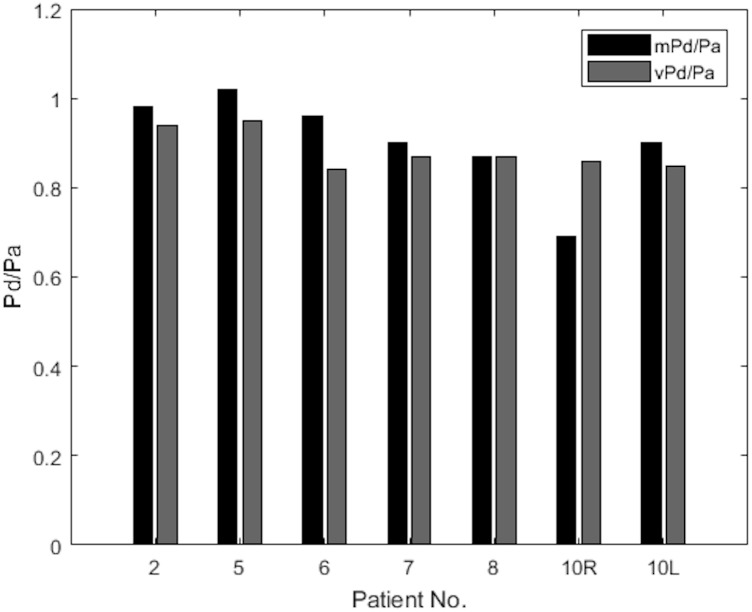
Measured versus virtual Pd/Pa.

The results of mPd/Pa and vPd/Pa are presented in parallel in the bar plot diagram of **Figure 6**. This bar plot illustrates mPd/Pa side-by-side with vPd/Pa for each patient, indicated by their number (Patient 10’s RAS on both renal arteries is distinguished with R for the right, and L for the left RAS).

**FIGURE 7:**
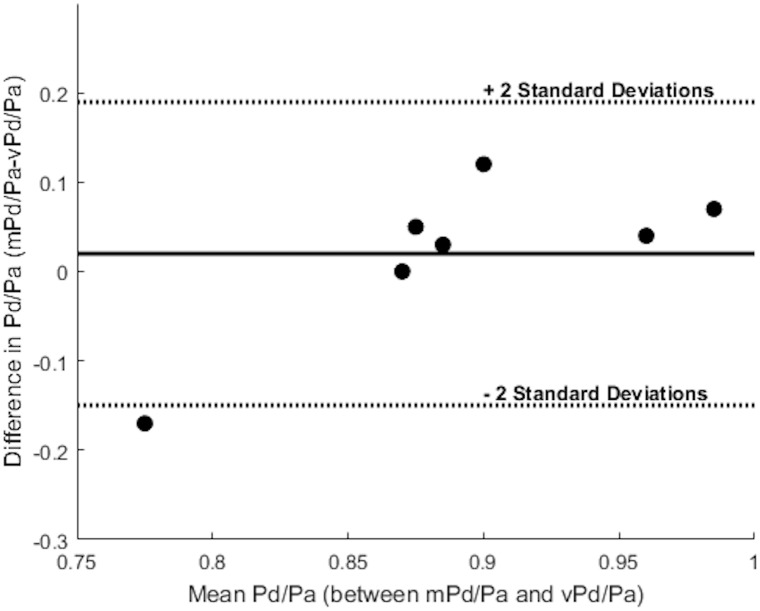
Bland Altman plot. The solid line represents the mean value of the difference between mPd/Pa and vPd/Pa, and the dotted lines create boundaries for ± 2 standard deviations from the mean difference. Each dot represents a stenosis and combines the knowledge for the difference and the mean value between the measured and the virtual calculations.

**FIGURE 8:**
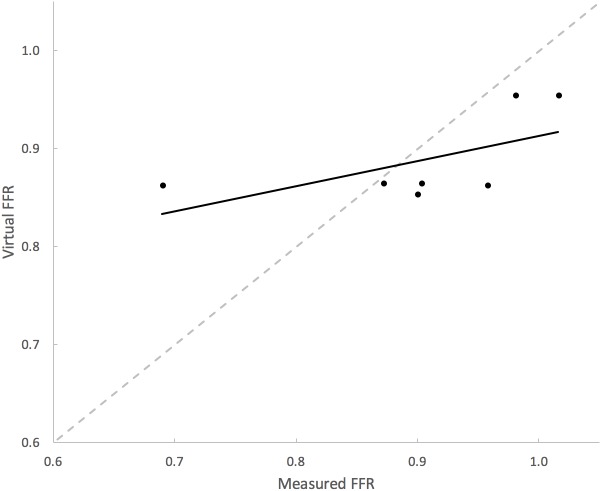
Correlation between mPd/Pa and vPd/Pa. The diagram plots mPd/Pa against vPd/Pa for each stenosis, where the segmented line in gray represents the points of exact agreement between measured and virtual calculations, while the solid line represent the data trendline of best-fit.

**Table 3** summarizes the measures of accuracy of our vPd/Pa estimations against mPd/Pa: the mean difference between mPd/Pa and vPd/Pa was ± 0.015, with an average absolute error of ± 0.064, representing a percentage error of 8.1%. Those quantitative measures are used to illustrate the accuracy of our computations, seen in the Bland Altman plot of **Figure 7**. The correlation between measured and virtual values (Pearson correlation coefficient *r* = 0.604) is illustrated in **Figure 8**.

**Table 3:** Quantitative accuracy of vPd/Pa.

Patient no.	mPd/Pa	vPd/Pa	Diameter stenosis (%)
2	0.981	0.954	46
5	1.016	0.954	42
6	0.958	0.862	43
7	0.903	0.865	53
8	0.872	0.865	76
10R	0.690	0.862	n.a.
10L	0.900	0.853	n.a.

**Measures of accuracy**
*Mean difference*		± 0.015	
*Standard deviation*		0.087	
*Mean absolute error*		± 0.064	
*Mean absolute error (%)*		8.1	
*Pearson’s coefficient (r)*		0.604	

*Quantitative comparison between mPd/Pa and vPd/Pa for our patient-specific data set, evaluating the mean difference between mPd/Pa and vPd/Pa, standard deviation, average absolute error, and Pearson’s correlation coefficient*.

### Diagnostic Accuracy

**Table 4** overviews the accuracy of our virtual simulations in identifying a physiologically significant RAS. Relative to mPd/Pa, the sensitivity, specificity, PPV and NPV of vPd/Pa was 1.0 (95% CI 0.4–1.0), 0.67 (0.13–0.98), 0.8 (0.3–0.99) and 1.0 (0.2–1.0). Overall diagnostic accuracy was 86%.

**Table 4:** Diagnostic accuracy of vPd/Pa.

Patient no.	Test outcome
2	True negative
5	True negative
6	False negative
7	True positive
8	True positive
10R	True positive
10L	True positive


*Diagnostic accuracy of vPd/Pa for our patient-specific data set on the basis of identifying a physiologically significant RAS*.

## Discussion

This proof-of-concept study investigates a novel *in silico* approach that uses CFD modeling to non-invasively estimate renal artery haemodynamics from routine CT imaging of RAS. Our results demonstrate that this approach is feasible and diagnostically accurate. This may have great value in reducing the need for invasive haemodynamic assessment. In this feasibility study, several challenges and limitations were encountered. Although the range of mPd/Pa reflects the expected clinical range, the sample size was modest. There were limited available data around 0.9 threshold and only one case was less than 0.8. It is likely that clinically and statistically more conclusive results might be achieved with a larger sample size.

The anatomy and geometry of the stenosed segments (**Figure 5**) are highly variable but some anatomic generalizations could be made: the arteries are tortuous, they emerge perpendicular to the aorta, and most of the stenoses are detected very close to the ostium of the renal artery, an observation which is clinically supported for ARASs (Kaatee et al., 1996). These anatomic observations also posed a challenge for the *in silico* simulations. The velocity field at the inlet of the renal artery will be influenced by these sharp bifurcating angles and there will be a difference when considering or not the aortic part. This different flow field might result in a different pressure distribution at these bifurcations that might influence adversely our vPd/Pa prediction across the stenosis. As part of this study we investigated the effect of including the aorta or not on the accuracy of vPd/Pa. We found that the difference in the vPd/Pa estimations was too small (< 3.8%) to justify the use of the aortic segment. Considering the added requirements on computational time, and boundary conditions that are largely unknown or will have to be invasively measured, it was decided that the workflow should use only the stenosed arterial geometry in the vPd/Pa calculations.

**FIGURE 9:**
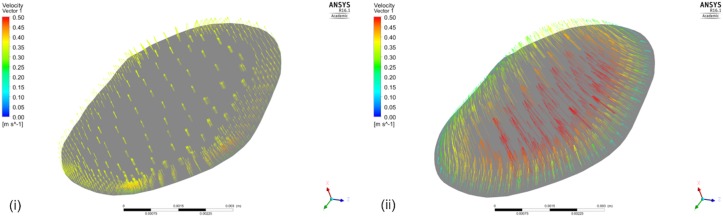
Velocity Vectors including and excluding the aorta. The figure shows the velocity vectors on a cross-sectional area of the renal artery close to the aorta for the simulation **(i)** that does not include the aortic geometry, and **(ii)** that does.

Executing the simulation including the aorta also contributed to an improved understanding of the expected velocity profile at the inlet of the renal artery. In our simulations, we assumed plug flow, i.e., the velocity was constant across the inlet cross-section (this is demonstrated in a cross-sectional area near the inlet in **Figure 9**(i) for the case excluding the aortic geometry). For the same cross-section in the case when the aortic geometry is included [**Figure 9**(ii)], the velocity profile upon entry to the renal artery, although not presenting plug flow, does not exactly represent a parabolic velocity profile either. At a later stage, a more complex velocity profile might be more suitable, supported by *in vivo* measurements.

For a subset of datasets (patient 7, patient 10L, patient 10R) we reported the potential impact of uncertainty in the location of data extraction on the predicted values of downstream pressure (**Table 2**). This showed only marginal effect on clinical significance but a direct correlation between data variability (standard deviation) and anatomical complexity, highlighting the importance for more precise protocols for more quantitative comparisons and analyses.

Even though patient-specific arterial geometries were used in the CFD calculations, the applied boundary flow conditions were generic (defined for healthy and young individuals) for renal flows, which might have compromised the precision of vPd/Pa. The potentially unrealistic high flow rates due to the generic boundary flow conditions might have led to the observed overestimation of vPd/Pa for all but one presented cases (**Figure 8**). However, echo Doppler measurements of maximum velocity at the stenoses were available, and these data were used and compared with velocity at the same locations, to validate the approach of using typical renal artery flows.

Despite the sample size and the generic boundary conditions, vPd/Pa for the present data set, showed a good agreement with the values of mPd/Pa (only Patient’s 10 Right RAS was evaluated just outside the 2 standard deviation boundaries, shown in **Figure 7**). This vPd/Pa underestimation for Patient 10 possibly indicates the importance of patient-specific boundary conditions, especially for cases of severe stenosis. Nonetheless, contrary to previous CFD studies (Morris et al., 2017), we can argue that the influence of geometry in our workflow seems more important than boundary conditions. It should also be noted that based on the estimated diagnostic accuracy, and in comparison with the standard anatomical criteria in **Table 1**, our workflow managed to evaluate only one false result in identifying a physiologically significant RAS (**Table 4**), demonstrating a very encouraging result for RAS diagnosis using physiology based metrics.

The aim of these computational simulations is achieving accuracy in calculating vPd/Pa but computational time and memory are also of importance. It should be noted that, alongside the presented steady simulations, finer mesh simulations and transient simulations over the heart cycle for the dataset were run, that rendered quantitatively insignificant differences in the resultant vPd/Pa.

**FIGURE 10:**
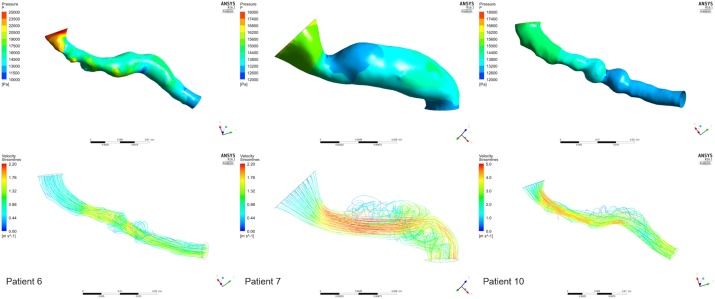
Examples from the CFD analysis. Pressure distributions (top row) and velocity streamlines (bottom row) are presented for the stenoses of three patients: 6 (left column), 7 (middle column), 10 (Right RAS, right column).

**Figure 10** presents the pressure distribution (top row) and velocity streamlines (bottom row) for three of our cases. Considering the complex geometries, the flow, as is evident in the figure, is also complex. Vortices are observed and areas of both high and low pressures can be identified. Estimating a Reynolds number for our data set is thus important. Reynolds number in the presented results ranged between 813 and 940, with two cases at 1463 and 1965. According to the theoretical Reynolds definition for internal flow in straight pipes, that indicates laminar flow. However, most of the presented geometries are far from an assumed straight cylinder which may induce turbulence at lower Reynolds number values. Therefore, consideration of turbulent effects in stenosed renal arteries might be further considered in the next research stage to achieve further accuracy.

Given the observations made in this study, there is a clear direction for the next stage of research. A larger clinical cohort, with a clinically representative range of mPd/Pa values will increase statistical confidence of the hypothesized correlations we demonstrated in this proof-of-concept study. Standardization and automisation of the workflow will be required in order to deal with a larger number of clinical cases. A further limitation was the use of retrospective CT angiographic and invasive haemodynamic data not specifically collected for the purposes of this study. Future studies are planned that will prospectively collect more detailed information of invasive haemodynamics and optimize CT or magnetic resonance image acquisition protocols. This will permit: (1) the standardization of image resolution for increased feasibility of volume segmentation, (2) the enhancement of *in silico* predictions by complementary non-invasive measures of renal artery blood flow using Doppler sonography (Li et al., 2008; Kaya, 2012), and (3) the more direct comparison of *in silico* with *in vivo* predictions by more precisely specifying the location of pressure measurements.

Information on the functionality of the post-stenosis tissue is important for the clinical diagnosis of RAS, but can also affect our computational simulations, as it is reflected on the value of peripheral resistance. eGFR which was measured in this study can provide such an assessment and although most of the provided cases presented normal levels of eGFR, biomarkers for kidney function could inform our *in silico* simulations in the future. Bearing in mind that there is no consensus in the medical community on a quantitative measure for the health of each individual kidney, creatinine clearance rates, kidney size, resistant hypertension, and velocity measurements comparing the stenosed artery with the aorta and the contralateral renal artery, have been previously examined to create an informed profile on stenosis severity (Zeller et al., 2008; Noory et al., 2016; Staub et al., 2016). There has also been recent research on the use of MRI for the assessment of kidney perfusion (Odudu et al., 2012). Consequently, knowledge on the functionality of the stenosed kidney provided clinically at a later study, combined with a statistically significant data set size, could contribute to the investigation of a correlation between peripheral resistance and renal function clinical indicators.

This study demonstrated the potential value of *in silico* assessment of renal haemodynamics for the purposes of RAS diagnosis, implementing a fast segmentation of high standard, and calculating reliable values of vPd/Pa. The next stage of research, based on the presented analysis, will largely contribute to reliably illustrating the importance of non-invasive renal haemodynamics on diagnosis.

## Conclusion

This is the first *in silico* assessment of renal artery haemodynamics from CT angiography. This approach is feasible and diagnostically accurate. Non-invasive renal artery haemodynamic assessment may facilitate precision medicine in patients with RAS and resistant hypertension.

## Author Contributions

AiM, AM, and PM conceived and designed the study. AO, JK, PK, AiM, and AM acquired and analyzed the clinical data. All authors analyzed and interpreted the study results and critically revised the article. AiM drafted the article. AM supervised the study.

## Conflict of Interest Statement

The authors declare that the research was conducted in the absence of any commercial or financial relationships that could be construed as a potential conflict of interest.

## Footnotes

1 https://clinicaltrials.gov/

2 http://www.aneurist.org//index.php
